# Frequently asked questions and answers (if any) in patients with adrenal incidentaloma

**DOI:** 10.1007/s40618-021-01615-3

**Published:** 2021-06-23

**Authors:** F. Ceccato, M. Barbot, C. Scaroni, M. Boscaro

**Affiliations:** 1 Endocrinology Unit, Department of Medicine DIMED, University of Padova, Via Ospedale Civile, 105-35128 Padova, Italy; 2 Endocrine Disease Unit, University-Hospital of Padova, Padova, Italy; 3 Department of Neuroscience DNS, University of Padova, Padova, Italy

**Keywords:** Adrenal incidentaloma, Autonomous cortisol secretion, Surgery, Multidisciplinary group

## Abstract

**Purpose:**

Adrenal incidentalomas (AIs) are incidentally discovered adrenal masses, during an imaging study undertaken for other reasons than the suspicion of adrenal disease. Their management is not a minor concern for patients and health-care related costs, since their increasing prevalence in the aging population. The exclusion of malignancy is the first question to attempt, then a careful evaluation of adrenal hormones is suggested. Surgery should be considered in case of overt secretion (primary aldosteronism, adrenal Cushing’s Syndrome or pheochromocytoma), however the management of subclinical secretion is still a matter of debate.

**Methods:**

The aim of the present narrative review is to offer a practical guidance regarding the management of AI, by providing evidence-based answers to frequently asked questions.

**Conclusion:**

The clinical experience is of utmost importance: a personalized diagnostic-therapeutic approach, based upon multidisciplinary discussion, is suggested.

## Introduction

Adrenal incidentalomas (AIs) are incidentally-found lesions during radiological investigations unrelated to adrenal-related disorders. The availability of imaging techniques, especially computed tomography (CT) and magnetic resonance (MR), enabled the increased detection of AIs over the years. In most cases, AIs are non-functioning benign formations (cortical adenomas) that do not require treatment, however they represent an important challenge for differential diagnosis among several benign and malignant pathologies, with a wide spectrum of endocrine activity (adrenal secretion is a continuum from non-functioning to overt-secreting forms) [1–4].

The prevalence of AIs, derived from autopsy data, is variable according to age: < 1% in young people, 3% in middle-aged adults and > 15% in subjects over 70 years old [5, 6]. Considering radiological studies, the prevalence in middle-aged subjects is 2–4%, up to > 10% in patients aged 70 or over. It is slightly more frequent in women, with a moderate prevalence on the right side [3]. In 10–15% of cases AIs are bilateral. As far as size is concerned, most authors consider “incidentaloma” only a lesion > 1 cm in diameter [2].

## Question 1: Is it malignant?

The first challenge for the endocrinologist is to promptly recognize a rare malignant form among the majority of benign lesions.

The adrenal glands have one of the greatest blood supply rates per gram of tissue [7]. Therefore, adrenal masses can be metastases of several primary tumors, especially if bilateral. Clinical history (positive for extra-adrenal malignancy) and patient’s presentation (age, rapid weight loss) should be ascertained before imaging. A recent population-based cohort study reported a 7.5% prevalence of metastasis in AIs, and that malignancy was 22 times more likely when the AI was discovered during cancer staging [8]. In patients with Colo-Rectal Cancer, the incidence of AIs was 10.5% (in 475 subjects); however, adrenal metastases could be ruled out in most cases (96%) with CT re-evaluation, follow-up imaging and multidisciplinary evaluation [9]. However, it is personal opinion of the authors that if an imaging study was performed during the evaluation of known extra-adrenal cancer, the AI does not fall into the definition of “incidentaloma”, because the imaging itself has been performed in a patient with an active malignancy, therefore the pre-test probability that the adrenal lesion represents a metastasis is high.

Morphological evaluation by CT and MR is of utmost importance to describe the shape and size of the lesion. Non-secreting cortical adenomas contain significant amounts of intracellular lipids. There is an inverse relationship between lipid concentration and the attenuation value obtained with un-enhanced CT: a signal intensity < 10 Hounsfield Units (HU) is suggestive of benign lipid-rich adenoma [2, 10, 11]. This approach fails to recognize up to 40% of benign adenomas (the so-called lipid-poor adenomas, with HU > 10) [12, 13]. A different un-enhanced CT attenuation value has been observed in patients with secreting adenoma: an overt cortisol secretion (defined with increased urinary cortisol excretion) may decrease the intracytoplasmic lipid droplets. 80% of cortisol-secreting adenomas have an un-enhanced attenuation value > 10 HU, and 65% ≥ 20 HU: doubling of the cortisol secretion is associated with a 4 HU increase [14]. In patients with subclinical cortisol secretion, unsuppressed serum cortisol after dexamethasone shows a positive correlation with adenoma diameters, morphologic parameters differ between secreting and non-secreting adenomas, either lipid-rich or lipid-poor [15]. In clinical practice, lipid-poor AIs are a heterogeneous group of tumors, and one third of those masses may have a borderline-malignant potential features, expressed by high Weiss or Lin-Weiss-Bisceglia score [16]. There is still debate whether the MR is better than the CT scan [2, 6, 17]. MR is used to characterize lipid-poor adenomas, especially when the appropriate methods (chemical shift among with in-phase and out-of-phase) are used [12].

In suspected cases, it is important to measure the wash-out time during enhanced CT: a wash-out > 60% in arterial phase or a relative washout > 40% in the delayed images (15 min for the venous phase) suggest a benign form with sensitivity of 82–97% and specificity of 92–100% [2, 18]. Azoury et al., in a retrospective study, considered patients who underwent unilateral adrenalectomy for an adrenal mass in 10 years (2005–2015): they achieved 100% specificity to predict adenomas considering benign features on preoperative CT (*n* = 143 with HU < 10, well-defined borders, homogeneous texture, absence of necrosis or calcifications, rapid washout on dynamic protocol) [19]. Recently, a European retrospective study collected attenuation values (HU) and absolute/relative washout in a cohort of patients with pheochromocytoma (PHEO). Only two out of 376 patients with PHEO presented a low attenuation value (< 10 HU), therefore Authors concluded that it seems reasonable to abstain from biochemical testing for PHEO in case of AI with an unenhanced attenuation value ≤ 10 HU [20].

**Fig. 1:**
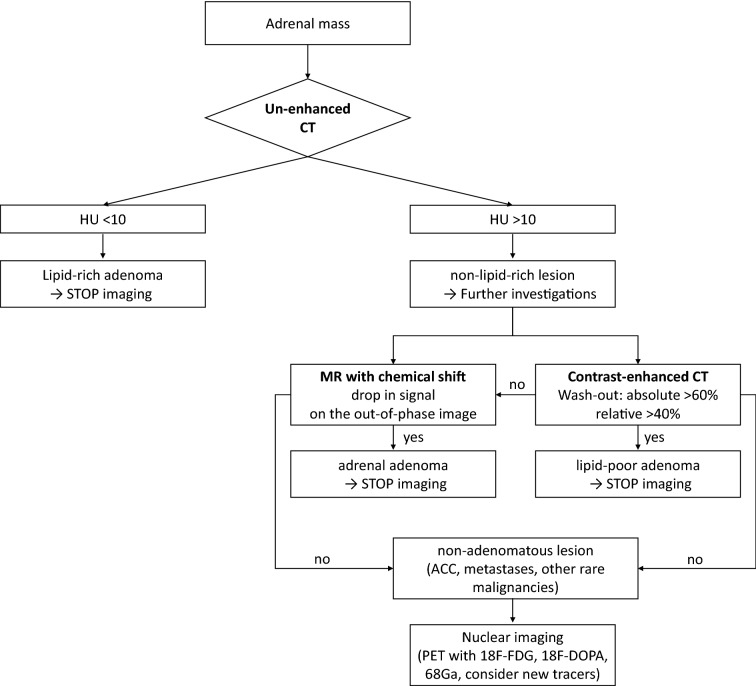
Suggested flow-chart at baseline visit in patients with adrenal incidentaloma. *CT* computed tomography, *HU* Hounsfield Units, *MR* magnetic resonance, *ACC* adreno-cortical carcinoma, *PET* positron emission tomography

At CT, the adenoma appears rounded, homogeneous, rich in lipids and with well-defined margins. Malignant lesions are often > 4–6 cm in diameter, present with irregular margins and texture, calcifications, necrosis and in some cases with invasion of surrounding structures. Regarding MR, the malignant forms are hypo-intense in the T1 and hyper-intense in the T2 images, showing an intense enhancement and a delayed washout. A T2 signal hyper-intensity (the light-bulb sign) is common in PHEO [10]. Nevertheless, a wide spectrum of imaging is reported: PHEOs may mimic other benign or malignant adrenal lesions [21]. As indicated in Fig. 1, we suggest contrast-enhanced CT or MR in case of suspected AI.

A 18–fluoro–2–deoxy–d–glucose (18F-FDG) positron emission tomography (PET) alone or combined with CT could be useful to characterize malignant forms. An adrenal mass is likely malignant when the uptake of 18-FDG is higher than that of the liver. However, false positives (sarcoidosis, tuberculosis, lipid-poor adenomas and PHEO) or negatives (necrotic-hemorrhagic areas present in malignant lesions) must be considered [22–26]. PET allowed to refine the diagnosis of pheochromocytoma by using 18F-meta-fluorobenzylguanidine, 18F-DOPA PET/CT, 18F-Fluorodopamine or 11C-hydroxyephedrine [27].

In addition to morphological diagnostic techniques, in case of indeterminate mass after an extensive biochemical and radiological assessment, a biopsy can be indicated in selected cases, after ruling out a PHEO. Nonetheless, adrenal biopsy presents low diagnostic value [28], a high-risk of cells spreading, and is not suggested in the study of AI [2].

**Fig. 2:**
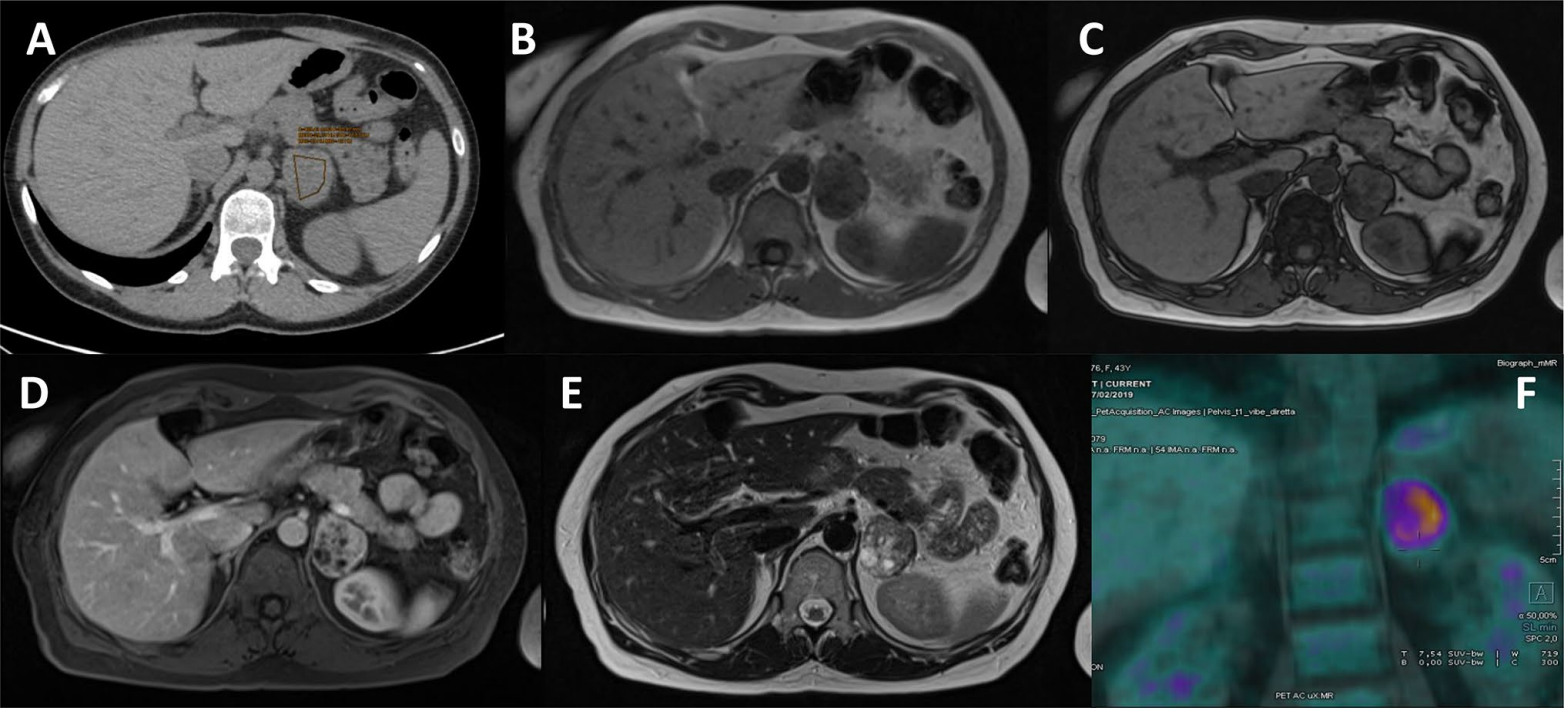
**a** Un-enhanced CT: right adrenal mass, 30 HU. **b**, **c **MR with chemical shift in-phase **b** and out-of-phase **c** without signal drop. **d**, **e** Enhanced MR with heterogeneous tissue and necrosis **f** 18-FDF PET with high uptake (SUV max 16). After surgery the histological examination was consistent with adrenal adenoma

In clinical practice, a close collaboration with an adrenal-dedicated radiologist is suggested, especially in a multidisciplinary team. In Fig. 2, we depicted a case discussed in the monthly multidisciplinary team dedicated to adrenal disease in Padova.

*Answer to question 1:* Malignancy must be assessed at the baseline visit in patients with AI. Patient’s presentation (age and history of extra-adrenal malignancy) and radiological evaluation (attenuation value > 10 HU in non-secreting mass, delayed washout, increased 18-FDG PET uptake) are markers of suspicion. In all suspected cases, a multidisciplinary discussion is suggested.

## Question 2: Is it hormonally active?

Considering AIs, non-secreting adenomas represent the largest part (70–80%), followed by glucocorticoid (CS), catecholamine (PHEO) and to a lesser extent mineralocorticoid secretion (Primary Aldosteronism, PA). Regardless of clinical aspect, the search for an increased endocrine production is generally carried out in all patients at baseline.

Considering cortisol excess, the hypothalamic–pituitary–adrenal (HPA) axis should be investigated in all patients presenting with an AI [2, 29–32]. Subclinical hypercortisolism does not present by definition any of the specific symptoms that characterize patients with overt Cushing’s syndrome (CS, as moon face, easy bruising, purple striae, cervical fat pad, and so on) [33]. Therefore, most authors preferred to use the term subclinical hypercortisolism, rather than subclinical CS or preclinical CS, because CS represents by definition a well-defined set of symptoms and the progression from non-secreting towards a clinical form is very rare [34]. These definitions have disappeared from the most recent Guidelines, replaced by autonomous cortisol secretion (ACS) [29]. The incidence of ACS is not well defined, because different diagnostic criteria have been adopted; it seems to be between 5 and 30% in most papers [35, 36], reaching even higher percentages in selected series [2, 3, 37, 38]. As a matter of fact, the lack of signs of CS or clinical features (hypertension, obesity, metabolic disorders, bone disease and so on) is not sufficient to define a non-functioning adenoma [37, 39].

The diagnosis of overt hypercortisolism (CS) is based upon first-line screening tests: urinary-free cortisol (UFC), dexamethasone suppression test (DST) and late-night salivary cortisol (LNSC) [33, 40]. To reduce the risks of false positives, and in agreement with many other authors, we consider a high likelihood of overt CS the alteration of at least two screening tests [2, 3, 29]. In case of overt cortisol secretion, low-suppressed ACTH levels are used to confirm adrenal origin [40, 41].

In normal subjects, a supra-physiological dose of glucocorticoids prompts the suppression of cortisol secretion: the DST explores the HPA axis negative feedback [42]. The overnight 1-mg DST is considered the most sensitive screening test in AI, even if there is still no complete agreement regarding cortisol cut-off. The Endocrine Society guidelines suggest to use the 50 nmol/L (1.8 μg/dL) cut-off to exclude overt hypercortisolism [31]. NIH and other scientific societies recommend the 138 nmol/L (5 μg/dL) to avoid false positives [2, 30, 32], also intermediate thresholds have been proposed (Chiodini et al. suggests 3 μg/dL as the best compromise [37]). The European Society of Endocrinology (ESE) Guidelines in collaboration with the Working Group of the European Network for the Study of Adrenal Tumors (ENS@T) consider ACS, amenable of surgical management in case of comorbidities, if cortisol after 1-mg DST is > 138 nmol/L, and possible ACS in the grey-zone 50–138 nmol/L (in such cases further biochemical tests to confirm cortisol secretion might be required, as UFC, LNSC or ACTH) [29]. At 50 nmol/L the sensitivity for CS is high (up to 100%), at the expense of a low specificity (< 85–90% [43, 44]). To add further insights, even patients with cortisol < 50 nmol/L after 1-mg DST might present some cortisol-related comorbidities, as increased insulin resistance and endothelial dysfunction [45]. The DST provide false positive results if cortisol binding globulin (CBG) levels are increased (as in case of estrogen therapy); on the other hand, falsely negative in case of hypoalbuminemia and low levels of CBG are reported. Moreover, inaccurate tests are not so rare, due to the interference of drugs that can accelerate or reduce the liver metabolism of dexamethasone [46]. To avoid these diagnostic pitfalls, combined cortisol and dexamethasone measurement after DST have been proposed [47–49].

ACTH, LNSC and UFC are used to assess HPA axis, however, they are conditioned by methodological issues. UFC is accurate in diagnosing overt CS, especially when measured with liquid chromatography tandem-mass spectrometry (LC–MS) [50, 51], however, it can be increased in physiologic/non-neoplastic hypercortisolism (obesity, depression, diabetes, pseudo-CS state [52–55]) and sometimes normal also in overt CS [50]. A poor diagnostic accuracy of LNSC to detect ACS has been documented, even if LC–MS methods are used [56–58]. Loss of circadian cortisol rhythm is a peculiar feature of overt CS, and LNSC should not be used in AI. ACTH measurement requires careful pre-analytical sampling and analytical procedures: commercially-available ACTH immunoassays are imprecise in the normal-low range (< 20 pg/mL) [59]: in selected cases, a human CRH test might unmask the ACTH responsiveness of ACTH-dependent hypercortisolism [60]. Moreover, also sensitivity of glucocorticoid receptor can affect the interpretation of first-line screening tests [61, 62]. Cortisol secretion is a continuum: no cut-off can unequivocally distinguish the physiological HPA axis from its abnormal activation (pseudo-CS) or overt CS. A post-surgical adrenal insufficiency may occur also in patients with “non-functioning” adrenal adenomas, because first line-screening tests can fail to recognize a mild cortisol secretion [63, 64]. The adrenal scintigraphy with ^131^I-19 norcholesterol should be considered to identify those patients who could benefit from adrenalectomy; however, it is of limited availability [65].

PA represents the most frequent form of endocrine hypertension. It affects 6% of the hypertensive population (up to 20% in selected cohorts). Patients with PA presents with suppressed renin, normal-elevated aldosterone concentration and hypokalemia in some cases [66]. Despite the relatively high prevalence of PA in the hypertensive population, its finding among AI is relatively low, ranging from 1.5 to 7% [2, 67]. Adrenal lesions of patients investigated with abdominal imaging in search of the causes of secondary hypertension should not considered “incidentalomas” [68]. Moreover, some guidelines recommend exploring mineralocorticoid secretion only in hypertensive patients with AI [2, 30], thus excluding those with normal/borderline blood pressure, in which an autonomous secretion of aldosterone could not be excluded a priori [69, 70]. It has been reported that forms of subclinical hyperaldosteronism are not so rare in patients with AI, especially in those with increased diastolic blood pressure levels [71]. Recent acquisitions have radically changed the classic division of the adrenal cortex into zones: clusters of aldosterone-producing cells are found in normal adrenals and close to the aldosterone-secreting tumors [72].

Aldosterone to renin ratio (ARR) is recommended by the Endocrine Society guidelines as the most reliable test for PA screening [66]. Serum potassium levels should be normalized and interfering drugs should be discontinued before ARR: all diuretics must be suspended for 4–6 weeks; angiotensin converting enzyme inhibitors, angiotensin receptor blockers and beta blockers require 2–4 weeks of withdrawal [66]. Recently, it has been reported that an early mineralcorticoid-receptor antagonists treatment did not seem to endanger the accuracy of ARR [73]. After the diagnosis of PA, confirmatory tests and PA subtyping (monolateral vs bilateral disease) are suggested [66], because a non-functioning AI may coexist with bilateral PA, in order to avoid an unnecessary adrenalectomy [74, 75].

PHEO represents about 7% of AIs as reported by the ESE-ENS@T guidelines [29], with an even higher prevalence for other authors [76], and a modern presentation of PHEO (despite the classic triad of headaches, sweating and palpitation) has been suggested in patients with AI [77]. With the currently available tests (metanephrines and accurate imaging), a diagnosis of PHEO should be rule-out at diagnosis in all patients with AI, especially before surgery [78].

Lenders et al. reported that 30% of PHEOs are incidental, and that the prevalence of the tumor is increasing over time [79]; these data are confirmed also in other series [80, 81], especially in large AIs. The clinical history reports hypertensive episodes only in a modest percentage of cases, therefore all guidelines suggest to test for PHEO all patients with AI [2, 29, 30, 32]. Assessment of plasma or urinary metanephrines levels are the screening test in a patient with suspected PHEO [82]. The diagnostic accuracy of metanephrines is higher than that of catecholamines, with reduced false positives, especially in the elderly. In this regard, recent data from Eisenhofer et al. offer specific age-related reference intervals for both plasma and urinary metanephrines [83]. The biochemical diagnosis of PHEO could be conditioned by numerous interfering factors: stress, increased sympathetic activity (myocardial infarction, stroke, hypoxia), the use of antidepressants, antipsychotics, beta blockers, antibiotics and glucocorticoid [82]. Moreover, also some foods (bananas, cheeses, coffee, yogurt, cured meats, soya sauce, red wine, fish, chocolate, figs, fava beans) should be avoided for 3–5 days before urinary collection [68].

The dosage of adrenal androgens, its precursors and metabolites in patients with AIs is commonly reserved for suspected cases of Congenital Adrenal Hyperplasia (often associated with normal-low cortisol and normal-high ACTH levels) [84, 85]. On the other hand, the androgens and adrenal sex hormones should be considered in case of rapid-onset hirsutism in female, because adrenocortical cancer (ACC) could be a rare but reasonable diagnosis [18].

An emerging approach is the measurement of urinary steroid metabolites: 24-h urine steroid excretion allow the identification of different steroid patterns, especially if measured with mass spectrometry [86]. In patients with PA, a glucocorticoid co-secretion is frequently found, contributing to metabolic risk [87] and ventricular hypertrophy [88]. The combination of CT and a panel of 15-steroid profile had the same accuracy of vein sampling to differentiate unilateral PA (amenable of surgical remission) from bilateral adrenal hyperplasia [89]. In case of ACTH-dependent CS there is a lower androgen precursors’ and a higher 11-dexoycorticosterone secretion than ACTH-dependent forms of hypercortisolism [90]. In patients with AIs, 21-deoxycortisol and 11-deoxycorticosterone after ACTH test were increased in ACS [91], and cortisol and corticosterone after 1-mg DST were associated with higher prevalence of severe-resistant hypertension and higher incidence of cardiovascular events [92]. An immature steroidogenesis is able to identify ACC with a specific fingerprint [93].

*Answer to question 2:* According to available literature, urinary metanephrines, ARR (after appropriate drug washout) and serum cortisol after 1-mg DST are suggested in all patients with an AI.

## Question 3: Is surgery an appropriate treatment in patients with ACS?

Surgery is the suggested therapy for AI in patients with secreting adenoma, suspicion of malignancy or in case of ACS and cortisol-related comorbidities (hypertension, impaired glucose metabolism, obesity, dyslipidemia, osteoporosis) in the ESE-ENS@T Guidelines [18]. It seems a clear and easy-to-manage indication; however, if the diagnosis of overt CS (with the full-blown complete clinical picture) could be plain, the appropriate detection of ACS and its cortisol-related comorbidities requires extensive clinical practice. There is a greater prevalence of cortisol-related comorbidities in patients with AI (either non-functioning or ACS), compared to normal control subjects [94–99]. Therefore, the reversal of cortisol-related comorbidities through the surgical management of ACS seems a reasonable choice.

Beneficial effects of adrenalectomy on cardiovascular risk in patients with ACS are not evidence-based, yet. From a clinical perspective, most patients are aged and have longstanding comorbidities, thus with limited improvement after surgery. On the other hand, the beneficial effect of surgery is based upon uncontrolled retrospective studies, where a selection-bias (versus conservative management in old or worst-prognosis patients) could not be ruled-out. Toniato et al*.* in one of the first prospective study described an improvement in metabolic and cardiovascular parameters after surgery [100], confirmed by other authors [101, 102]. In a systematic review, Iacobone et al. selected six retrospective and one prospective studies, including 230 patients: surgical treatment was able to normalize hypercortisolism, improving blood pressure, glucose metabolism and obesity in 72, 46 and 39% of patients, respectively, when compared with the conservative-treatment group [103]. Petramala et al. evaluated 70 patients with ACS: only those treated by unilateral adrenalectomy (*n* = 26) showed a significant decrease in blood pressure levels [104].

A metanalysis of 26 papers (584 patients) comparing surgery and conservative management in patients with ACS, reported that adrenalectomy improves arterial hypertension, diabetes, dyslipidemia and obesity [105]. However, the collection of heterogeneous studies resulted in a low-to-moderate level of evidence. In the meta-analyses, as in clinical practice, the criteria to define ACS were different. Moreover, not only the confidence intervals were large, suggesting a significant individual variability, but also inconsistent definitions of comorbidities and the degrees of improvement limited the final accuracy of the outcome. Even patients with non-functioning AI showed an improvement in blood pressure after adrenalectomy [106]. Most of the non-functioning AI remain asymptomatic for life, and the natural history of non-functioning or subclinical forms is still poorly understood and follow-up too limited [107, 108].

Taking into account that the main consequences of subclinical hypercortisolism are arterial hypertension, metabolic disorders and diabetes, the New Zealand group coordinated by Goh et al. suggests to evaluate case by case before engaging in too many investigations and wasting unnecessary resources [109]. We must keep in mind that in ACS the cortisol-related comorbidities, such as cardiovascular risk factors, impaired glucose or lipid metabolism, bone disease and the impaired quality of life [62, 110–115], are related to modest changes of cortisol secretion.

In a follow-up study (average of 7.5 years), Di Dalmazi et al*.* observed that in patients with stable/intermediate phenotype of hypercortisolism and in those with ACS the overall survival (considering all causes of death) was lower than in non-secreting AIs [96]. Similar results have been reported by De Bono et al.: mortality from cardiovascular and infectious diseases was increased in cases of ACS [97]. In light of these literature data, which have not been adequately considered by the latest ESE-ENS@T guidelines, Morelli et al*.* underline the need to follow all incidentalomas with adequate clinical and endocrine follow-up associated with a careful assessment of possible cardiovascular risks [116].

At present, there are no prospective randomized studies on cases of patients with functioning incidentaloma to undergo surgery or medical therapy (the drugs available are those reserved for overt CS), to define which is the best treatment.

To conclude, several factors must be considered before surgery in a patient with ACS (age, cortisol-related comorbidities [117, 118]) in a multidisciplinary team discussion, in order to identify those patients that would be most likely to benefit from surgery [18].

*Answer to question 3:* Surgery should be considered for patients with ACS and cortisol-related comorbidities. However, a comprehensive multidisciplinary discussion is suggested, in order to balance the benefit and the risk of surgery.

## Question 4: Can a non-functioning lesion become ACS and a benign adenoma become malignant?

The natural history of AI is not fully reported: there is few evidence that non-functional and benign cortical adenomas can become functional, or even malignant [4, 119].

In a retrospective study, Pantalone et al. showed that an absolute increase of 0.8 cm, an annual growth of 0.64 cm or an increase in size > 25% are indicative of malignancy [120]. However, malignancy is reported also in cases without any significant growth in 3–16 months of follow-up [120]. In a meta-analysis of over 1000 patients with benign AI, two cases of malignancy were reported after 2–4 years [6]. Similar results can be found in a Turkish study: only one malignant node was found among 277 patients followed for 2 years (the adrenal mass increased from 24 to 84 mm in diameter in 6 months [121]). In a 4-year follow-up study of 77 patients, an increase > 0.5 cm has been observed in 30% and > 1 cm in ten cases; malignancy was not observed, four increased masses become functioning; a reduction was reported in six adenomas [122]. No evidence of malignancy or hyper-function has been reported in a Swedish 2-year follow-up study involving 126 patients [123]. Several studies reported the development of an overt secretion in up to 11% of cases [1, 121, 122], especially in masses > 3 cm [124]. Morelli V. et al.in a retrospective study of 206 patients concluded that masses > 24 mm in diameter present high-risk to develop ACS [125].

The meta-analysis of Cawood et al., including data collected between 1980 and 2008, reports an increase in size in 15% of AIs and a new-onset of hyper-function in 1% [4]. According to these data, it was subsequently suggested to limit the follow-up. Therefore, unlike the previous guidelines [30] which suggested a radiological control every 6–12 months for 1–2 years and then annually for 5 years, the ESE-ENS@T guidelines do not foresee any follow-up for smaller incidentalomas (< 4 cm), with benign features (< HU at CT) and non-functioning behavior at the baseline evaluation [18, 126].

A group of Finnish researchers, hypothesizing that a lipid-rich adenoma (HU < 10) requires a radiological follow-up after 5 years, described 54 patients with AI (< 4 cm) and with normal functional tests (two cases of hyperfunction). After 5 years the size of the lesion remained unchanged (from 19 ± 6 mm to 20 ± 7 mm), as well as the function. Only one case showed 8 mm growth, surgery confirmed a cortical adenoma [127]. A group of Korean researchers retrospectively tested 1149 patients (mean age 54 years) collected between 2000 and 2013 in a single center [119], according to the ESE-ENS@T guidelines 68% had non-functioning AI. To distinguish between benign and malignant masses it was calculated that the mass cut-off should have a diameter of 3.4 cm (with a sensitivity of 100% and specificity of 95%) and a HU value at CT of 19.9 (with sensitivity of 100% and specificity of 67.4%). Most of the non-functioning masses showed no change in size during 4 years of follow-up [119]. Considering the cut-off of 50 nmol/L after 1 mg DST, 28% of non-functioning AI developed an ACS during follow-up, no progression to an overt CS was observed.

Finally, in a recent study from New Zealand, 101 patients were followed for 3-years. Growth was observed in 5% of cases, malignancy was not documented. At the beginning of the study nine cases showed signs of cortisol excess. At 3 years, five of the nine cases of hyperfunction normalized, while the other five showed a subclinical cortisol secretion (5%) [109].

Since no cases of hyperaldosteronism have been reported during the numerous follow-up studies, it is quite common practice not to control the mineralocorticoid function after baseline evaluation. Also anecdotal cases of PHEO have been reported in previous studies with less accurate diagnostic tests [120–122, 124].

*Answer to question 4:* The progression to a malignant form is anecdotal. In some patients a non-functioning AI could evolve in ACS, therefore 1-mg DST is suggested in the follow-up.

## Question 5: Is perioperative period a matter of concern?

Surgery of functioning adrenal tumors remains at a higher risk for comorbidities, also in patients with ACS, despite continuous evolution, technical improvements and reduction of perioperative risks. There are no evidence-based protocols, probably due to the heterogeneity of the cases and the limited number of patients reported.

**Fig. 3:**
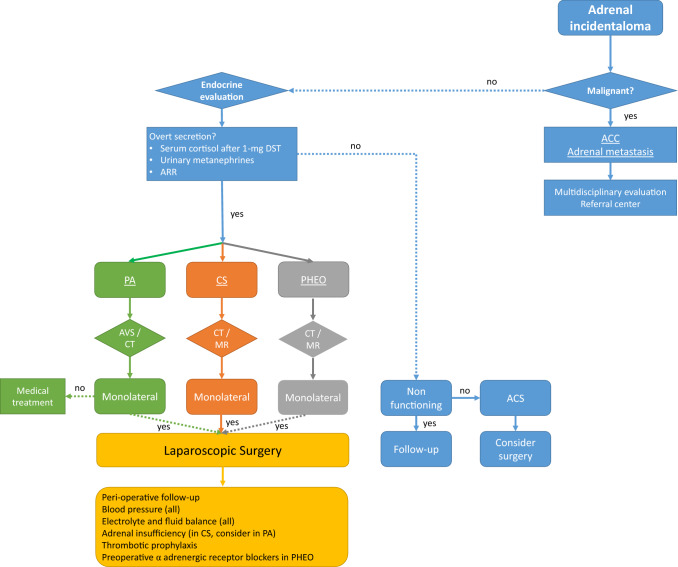
Suggested management of adrenal incidentaloma. *ACC* adrenocortical cancer, *PA* primary aldosteronism, *AVS* adrenal vein sampling, *CS* Cushing’s syndrome, *ARR* aldosterone to renin ratio, *PHEO* pheochromocytoma, *DST* dexamethasone suppression test, *CT* computed tomography, *MR* magnetic resonance

Laparoscopic surgery, well-tolerated and accompanied by less morbidity, is effective in normalizing cortisol levels and reducing cortisol-related morbidity [128, 129]. Perioperative management is critical, not only for overt CS [130]: patients with ACS may present cortisol-related comorbidities that could increase surgical risk [110, 131, 132]. After adrenalectomy, replacement therapy with hydrocortisone is mandatory in CS, and should be considered also in patients with ACS, to avoid post-surgical adrenal insufficiency [64, 133]. HPA axis inhibition must be taken into account even in case of ACS or non-functioning incidentalomas, therefore blood pressure, serum glucose and sodium levels must be assessed in the perioperative period, as suggested in Fig. 3. The correct diagnosis of HPA axis before surgery is able to predict post-surgical adrenal insufficiency: 90% of patients diagnosed as cortisol-secreting according to strict criteria (DST + 2 screening tests) need replacement therapy against only 50% of cases diagnosed with DST alone [134]. Cortisol levels > 83 nmol/L after DST, increased UFC and ACTH < 10 pg/mL are the best parameters for predicting post-surgical hypoadrenalism [135].

In patients with PA, the normalization of blood pressure and potassium levels should be considered before surgery [136, 137]. Post-intervention data show changes in renal sodium reabsorption, vasodilatation of the arterioles, structural changes of the renal parenchyma and, in case of long-lasting PA, suppression of mineralocorticoids of the residual adrenal gland [138]. A subset of patients with PA have cortisol co-secretion [87], thus requiring glucocorticoid treatment.

The treatment of choice for PHEO is adrenalectomy [82, 139]. Surgery can be complicated by the sudden release of catecholamines due to anesthesia induction or manipulation of the adrenal mass, moreover, the tumor can be silent [81]. All patients with PHEO needs adequate preparation before surgery with α-adrenergic receptor blockers (2 weeks) and hydration (immediately before surgery), reducing the perioperative morbidity from 40 to 7% [140].

*Answer to question 5:* A close cooperation between endocrinologists and surgeons is mandatory: endocrine management of perioperative period is of utmost importance. Glucocorticoid substitutive treatment must be considered in case of cortisol secretion (either overt CS or ACS).

## Question 6: Is a different management required for bilateral adrenal incidentaloma?

The usual presentation of bilateral adrenal incidentalomas (BAI, up to 9–17% of AI) is that of two discrete cortical adenomas, one on each adrenal; in rare cases, both adrenals may be massively enlarged by the presence of multiple macronodules (> 1 cm), termed Primary Macronodular Adrenal Hyperplasia (PMAH). In BAI with PMAH, the concept of ACTH-independent cortisol secretion was abandoned after the demonstration of paracrine and autocrine adrenal ACTH secretion [141].

The various etiologies of unilateral or bilateral AI vary depending on whether recruitment was performed in an endocrine or surgical setting: non-secreting AI are prevalent in the former, while ACC and secreting tumors are over-represented in the latter [3, 142, 143]. Adrenal metastases are often bilateral, on the contrary bilateral ACC is a rare condition [144]. In clinical practice, bilateral masses do not necessarily represent the same entity on both adrenals: the coexistence of different adrenal diseases is not uncommon, and in selected cases nodular adrenal hyperplasia could be ACTH-dependent, as in Cushing’s Disease [145] or Congenital Adrenal Hyperplasia [84].

The initial evaluation of BAI should aim to assess malignancy and functional status. Regarding imaging, each lesion should be assessed separately [29]. Benign lesions are lipid-rich (attenuation value < 10 HU), in case of lipid-poor adenomas an enhanced CT or a MR is able to further characterize the mass.

The endocrine assessment is similar to that of unilateral AI. The most common endocrine disease is cortisol excess (ACS), which is more prevalent in BAI. The prevalence of metabolic cortisol-related comorbidities (obesity, diabetes, hypertension, dyslipidemia) is similar to those patients with unilateral AI [146, 147]; however, only fracture risk seems increased in BAI [148].

Bilateral non-adrenal malignancies (metastasis, adrenal lymphoma [149]) may lead to adrenal insufficiency due to neoplastic infiltration of the adrenal glands: in selected cases, especially if imaging is not conclusive for lipid-rich AI and ACTH levels are increased, an insufficient cortisol secretion should be reasonably suspected.

In patients with PMAH and cortisol secretion, a special relevance is the study of illegitimate membrane G-protein coupled receptors (GPCRs). The activation of these GPCRs activate the cyclic-AMP/protein kinase A (PKA) signaling, leading to the transcription of steroidogenic factors, acting as ACTH [142]. These receptors could be ectopic or eutopic (over-expressed), and are studied applying various stimuli with dynamic tests (as meal test, upright posture test, luteinizing hormone-releasing hormone or metoclopramide test), to document a paradoxical cortisol secretion [150]. In case of aberrant receptors, an attempt to control cortisol hypersecretion could be performed with octreotide, propranolol, long-acting gonadotropin-releasing hormone agonist or other selected receptor agonists or antagonists [144, 151].

Bilateral adrenal masses, along with cortisol secretion and the documentation of familial cases, suggests a genetic predisposition. Recently, Armadillo repeat containing 5 (ARMC5) mutations have been described in familial cases and in sporadic patients with CS [152, 153]. Although ARMC5 mutations reduce the steroid secretory capacity of each cell, the overall cortisol secretion is increased due to massive enlargement of the adrenals [154–156]. In 2015, the association between ARMC5 and the presence of meningiomas has been described [157].

In patients with PMAH and cortisol secretion, the treatment of choice (surgery or medical therapy with steroidogenesis inhibitors) is not evidence-based, therefore a multidisciplinary evaluation in a referral center is suggested.

*Answer to question 6:* The assessment of malignancy and functional status in bilateral AI is similar to that of unilateral lesion. However, PMAH and ARMC5 mutations should be considered in case of overt cortisol secretion.

## Conclusions

Several issues, summarized in Table 1, are still debated in patients with AI.

**Table 1:** Open issues in patients with AI

Evaluation	Remarks and open issues
Urinary metanephrines	All patients (according to guidelines) Only AI with HU > 10?
ARR	All patients? Only those with hypertension or spontaneous hypokalaemia?
Serum cortisol after 1-mg DST	All patients (according to guidelines) High rate of “inadequate” response (cortisol < 50 nmol/L) → requiring further examinations for cortisol-related comorbidities (surgery or conservative management)
Late night salivary cortisol	Only if clinical features of overt CS
Urinary free cortisol	After 1-mg DST to rule out CS?
Un-enhanced CT	All patients, look for attenuation value (HU < 10)
Contrast-enhanced CT or MR	Only if HU > 10 in unenhanced CT
Follow-up	According to basal CT, endocrine function and clinical history AI could evolve in overt cortisol secretion → repeat 1-mg DST in the follow-up urinary metanephrines and ARR only in selected cases (i.e. worsening hypertension)
Surgery for AI	Only in selected cases with ACS and cortisol-related comorbidities (awaiting for evidence-based data)
Peri-operative management	Substitutive glucocorticoid treatment in all cases of cortisol secretion (overt CS and ACS) after surgery Careful management of blood pressure and sodium/potassium in PA and PHEO

As a matter of fact, is difficult to define a rigid protocol in the absence of prospective studies in patients with AI, as well as lacking of prognostic factors to predict morpho-functional modifications of the mass. Without these elements, the hormonal studies, the type of imaging, the plan of follow-up and its duration must be based on the characteristics of the lesion (size, morphology, unilateral, bilateral), the age and gender of the patient and the presence of comorbidities (active cancer, cardiovascular diseases, high blood pressure, diabetes, osteoporosis etc.).

The clinical experience is of utmost importance: a personalized diagnostic-therapeutic approach, based upon multidisciplinary discussion, is suggested.

## Data Availability

Not applicable in a review paper.
